# A Theoretical Multifractal Model for Assessing Urea Release from Chitosan Based Formulations

**DOI:** 10.3390/polym12061264

**Published:** 2020-06-01

**Authors:** Manuela Maria Iftime, Stefan Andrei Irimiciuc, Maricel Agop, Marian Angheloiu, Lacramioara Ochiuz, Decebal Vasincu

**Affiliations:** 1 Romanian Academy of Sciences, Petru Poni Institute of Macromolecular Chemistry, 41A Grigore Ghica Voda Alley, 700487 Iasi, Romania; ciobanum@icmpp.ro; 2 National Institute for Laser, Plasma and Radiation Physics—NILPRP, 409 Atomistilor Street, 077125 Bucharest, Romania; stefan.irimiciuc@inflpr.ro; 3 Department of Physics, “Gh. Asachi” Technical University of Iasi, 700050 Iasi, Romania; m.agop@yahoo.com; 4 Center for Services and Research in Advanced Biotechnologies, Calugareni, Sanimed International Impex SRL, Road Bucuresti-Magurele, no. 70 F, sector 5, 077125 Bucharest, Romania; marian.angheloiu@sanimed.ro; 5 University of Medicine and Farmacy Grigore T. Popa Iasi, 700115 Iaşi, Romania; deci_vas@yahoo.com

**Keywords:** chitosan, multifunctional materials, multifractal theoretical model

## Abstract

This paper reports the calibration of a theoretical multifractal model based on empirical data on the urea release from a series of soil conditioner systems. To do this, a series of formulations was prepared by in situ hydrogelation of chitosan with salicylaldehyde in the presence of different urea amounts. The formulations were morphologically characterized by scanning electron microscopy and polarized light microscopy. The in vitro urea release was investigated in an environmentally simulated medium. The release data were fitted on five different mathematical models, Korsmeyer–Peppas, Zero order, First order, Higuchi and Hixson–Crowell, which allowed the establishment of a mechanism of urea release. Furthermore, a multifractal model, used for the fertilizer release for the first time, was calibrated using these empirical data. The resulting fit was in good agreement with the experimental data, validating the multifractal theoretical model.

## 1. Introduction

In recent years, fertilizer release has become an important topic in the field of agriculture. With advances in material design and engineering, new multifunctional materials have been introduced for the development of soil conditioners, particularly in fertilizer delivery systems [1]. Hydrogels are an important class of materials suitable for this purpose; they have substantial applicability in various domains such as medicine, agriculture, food industry, water treatments and so on [2]. Hydrogels obtained from both natural and synthetic macromolecules were extensively used as a matrix for controlled drug release with the aim to maximize the bio-efficacy, simplify clinical applicability and improve quality of life [2,3]. In recent years, the concept of hydrogel matrix has been translated to agriculture, being used as a matrix for different fertilizers aiming to increase their efficiency by controlled release [4]. Among the hydrogels, those obtained from renewable resources such as chitosan, present suitable properties which make them very important for delivery systems. They are biocompatible and biodegradable, and they present antifungal and antiviral activity [5]. Moreover, the hydrogels can swell and keep the moisture in soil for a longer time, and have the ability to encapsulate fertilizers by strong chemical or physical forces, further favoring their release in a controlled prolonged manner [2].

The beneficial properties of the chitosan hydrogels can be further improved by a proper choice of the crosslinker [5,6]. In this context, our group succeeded in preparing chitosan hydrogels by crosslinking with nontoxic monoaldehydes, some of them of natural origin [7,8,9,10,11,12,13]. The advantage of such a method proved to be the easy tuning of the hydrogel properties by an appropriate choice of the aldehyde. Accordingly, the use of salicylaldehyde led to biodegradable and biocompatible hydrogels with excellent mechanical, swelling and self-healing properties [9]. Taking into consideration these particular properties, the system was further investigated as a multifunctional matrix capable of releasing the fertilizer in a controlled manner [14]. As model fertilizer, urea was used, considering its high nitrogen content and low cost and also the possibility to improve its efficiency by minimizing loss by volatilization, denitrification or leaching processes [15]. By varying the crosslinking density and the urea amount, a large series of formulations was prepared, and the prolonged release ability was investigated. It was proved that these systems are promising soil conditioners, which deserves deeper investigation into the morphology–release behavior relationship for a better understanding of the mechanisms which govern the urea release, to allow further improvements of the design.

It is known that the fertilizer release from different matrix polymers is affected by multiple complex factors, such as the matrix structure, which further influences the swelling capacity and degradation, the release medium (pH, temperature, ionic strength) and the possible interaction between the fertilizer and carrying matrix [16]. Consequently, for a better understanding of the urea release mechanism from the salicyl-imine-chitosan matrix, we propose to assess the fertilizer release kinetics using both empiric and multifractal type laws.

## 2. Materials and Methods

### 2.1. Materials

Chitosan of low molecular weight (314 kDa, DA = 87%), salicylaldehyde of 98% purity, urea of 98% purity, ethanol, and glacial acetic acid were purchased from Aldrich and used as received. Bidistilled water was obtained in our laboratory.

### 2.2. Preparation of the Urea Release Systems

The formulations used in this paper as urea release systems were prepared according to a procedure mainly based on the in situ encapsulation of urea during the hydrogelation process of chitosan with salicylaldehyde (SA) [14]. By varying the molar ratio between amino groups of chitosan and aldehyde groups of salicylaldehyde, and the amount of urea, a series of 8 formulations was prepared (Table 1). The urea amount was calculated to be half, equal or double compared to the matrix amount, to give a final content in the formulation of 0%, 33%, 50% and 66% w/w (Table 1). The formulations were obtained as hydrogels, which were further lyophilized to give the dry formulations in the form of xerogels, which were used for investigations. They were coded 1.5-Ux and 2-Ux, where the 1.5 and 2 numbers reflect the molar ratio between the functional groups (NH_2_/CHO = 1.5/1 and 2/1, respectively) and x in Ux indicates the mass ratio of the urea to the blank matrix, giving a different percent of urea in different formulations. The 1.5-U0 and 2-U0 samples represent the blank matrix, without urea, which were used as references. Table 1 presents the amounts of the reagents used for the preparation of the urea release systems and their codes.

### 2.3. Methods

The xerogels formulations were obtained by the lyophilization of corresponding hydrogel formualtions using Labconco FreeZone Freeze Dry System equipment, for 24 h at −54 °C and 1.512 mbar.

The morphology of the formulations was investigated on the corresponding xerogels, using a field emission Scanning Electron Microscope (SEM) EDAX–Quanta 200 at accelerated electron energy of 20 KeV.

The supramolecular ordering of the xerogels formulations was observed with a polarized light microscopy (POM) with a Leica DM 2500 microscope.

### 2.4. The In Vitro Urea Release Protocol

The in vitro urea release was investigated for 35 days, at room temperature, using distilled water as the release medium. For a proper comparison, the amounts of formulations used in this investigation were previously weighted to contain the same amount of urea (50 mg). The formulations were immersed into vials containing 10 mL of distilled water. At fixed intervals, at each hour on the first day, and on each day over the next 35 days, 1 mL of supernatant was withdrawn from the vials and replaced with 1 mL of distilled water. The supernatant samples (1 mL each) were collected, lyophilized and the quantity of released urea was measured by ^1^H-NMR spectroscopy, by fitting on a calibration curve [14]. The proton spectra were recorded on a Bruker Avance NEO 400 MHz spectrometer equipped with a 5 mm broadband inverse detection z-gradient probe. Chemical shifts were described in δ units (ppm) and were referenced to sodium 3-(trimethylsilyl)-[2,2,3,3-d4]-1-propionate (TSP) as external standard at 0.0 ppm. The experiments were performed in duplicate. The calibration curve was realized by graphical representation of the integral value of urea protons vs. concentration, as obtained for 8 urea solutions in dimethyl sulfoxide-d_6_ (DMSO-d_6_) of known concentration. For the NMR study of urea released from samples, the certain quantities (1 mL) of supernatant were lyophilized and then dissolved in 0.6 mL DMSO-d_6_. The obtained solutions were transferred in NMR tubes containing capillaries with known concentrations of TSP in D_2_O.

### 2.5. Evaluation of the Release Kinetics

In order to investigate the mechanism of the fertilizer release, the release data of the studied formulations were fitted on the 5 different mathematical models: Korsmeyer–Peppas, Zero order, First order, Higuchi and Hixson–Crowell [17,18]:

- *Zero order model*: *Q_t_ = k_o_·t*, where *Q*_t_ is the amount of urea dissolved in the time *t* and *K*_0_ is the zero order release constant.
- *First order model*: *logQ_t_ = k·t/2.303*, where *Q*_t_ is the amount of urea released in the time *t* and *K* is the first order release constant.
- *Korsmeyer–Peppas model*: *M_t_/M_∞_ = K·t^n^,* where *M*_t_/*M*_∞_ is the fraction of urea released at the time *t*, *K* is the release rate constant and n is the release exponent.
- *Higuchi model*: *Q_t_ = k_H_·t^1/2^*, where *Q*_t_ is the amount of urea released in the time *t* and *K*_H_ is the Higuchi dissolution constant.
- *Hixson–Crowell model*: *Wo^1/3^-Wt^1/3^ = k·t,* where *W*_0_ is the initial amount of urea in the formulation, *W*_t_ is the remaining amount of urea in formulation at time *t* and *K* is a constant.

### 2.6. Theoretical Model

Our fundamental hypothesis is that the structural units’ dynamics in the polymer–drug complex systems are described by continuous but non-differentiable curves (multifractal curves). In such a context the drug release dynamics will be described through the multifractal theory of motion in the form of hydrodynamic regimes at various resolution scales (multifractal hydrodynamic model [19,20,21,22,23,24]).

Therefore, let us consider one-dimensional multifractal hydrodynamic equations S (18) and S (19) from Supplementary Material:

(1) $$\partial_{t}V_{D}+V_{D}\partial_{x}V_{D}{}^{}=-\partial_{x}\left[-2\lambda^{2}{\left(dt\right)}^{\left(\frac{4}{f\left(\alpha \right)}\right)-2}\frac{\partial_{x}\partial_{x}\sqrt{\rho }}{\sqrt{\rho }}\right]$$

(2) $$\partial_{t}\rho +\partial_{x}\left(\rho V_{D}\right)=0$$

In Equations (1) and (2) *V_D_* is the differentiable velocity, ρ is the state density, λ is a coefficient associated to the multifractal-non-multifractal transition, *dt* is the scale resolution, t is the nonfractal temporal coordinate and the affine parameter of the movement curve, x is the spatial fractal coordinate and f(α)  is the singularity spectrum of fractal dimension [21,22,23,24].

These equations for initial and boundary conditions [19,20]:

(3) $$V_{D}\left(x,t=0\right)=V_{0},\rho \left(x,t=0\right)=\frac{1}{\sqrt{\pi }\alpha }\exp\left[-{\left(\frac{x}{\alpha }\right)}^{2}\right]$$

(4) $$V_{D}\left(x=V_{0}t,t\right)=V_{0},\rho \left(x=-\infty ,t\right)=\rho \left(x=+\infty ,t\right)=0$$

with *V*_0_ the initial velocity and *α* the parameter of the gaussian distribution of positions, using the mathematical procedure from [25,26,27,28], provide the following solution:

(5) $$V_{D}\left(x,t,\sigma ,\alpha \right)=\frac{V_{0}\alpha^{2}+{\left(\frac{\sigma }{\alpha }\right)}^{2}xt}{\alpha^{2}+{\left(\frac{\sigma }{\alpha }\right)}^{2}t^{2}}$$

(6) $$\rho \left(x,t,\sigma ,\alpha \right)=\frac{{\left(\pi \right)}^{-1/2}}{{\left[\alpha^{2}+{\left(\frac{\sigma }{\alpha }\right)}^{2}t^{2}\right]}^{1/2}}exp\left[-\frac{\left(x-V_{0}t\right)}{\alpha^{2}+{\left(\frac{\sigma }{\alpha }\right)}^{2}t^{2}}\right]$$

with

(7) $$\sigma =\lambda {\left(dt\right)}^{\left[\frac{2}{f\left(\alpha \right)}\right]-1}$$

Introducing the non-dimensional variables

(8) $$\frac{x}{V_{0}\tau_{0}}=\xi ,\;\;\frac{t}{\tau_{0}}=\eta$$

and non-dimensional parameters

(9) $$\frac{\sigma \tau_{0}}{\alpha^{2}}=\mu ,\;\;\frac{\alpha }{V_{0}\tau_{0}}=\phi$$

with $\tau_{0}$ the specific time, Equations (5) and (6) become

(10) $$V_{D}\left(\mu ,\xi ,\eta \right)=\frac{V_{D}\left(x,t\right)}{V_{0}}=\frac{1+\mu^{2}\xi \eta }{1+\mu^{2}\eta^{2}}$$

(11) $$\rho \left(\mu ,\xi ,\eta \right)=\pi^{1/2}\;\alpha \rho \left(x,t\right)={\left(1+\mu^{2}\eta^{2}\right)}^{-1/2}\exp\left[-\frac{{\left(\xi -\eta \right)}^{2}}{\phi^{2}\left(1+\mu^{2}\eta^{2}\right)}\right]$$

In such a context, since the state density (ρ(μ,ξ,η)) defines the number of structural units in the polymer–fertilizer complex system and considering that $\bar{m}$ is the non-dimensional rest mass of the polymer–fertilizer structural units, then the non-dimensional mass variation (with respect to non-dimensional time η of the fertilizer release mechanism $\frac{d\bar{M}}{d\eta }$) is represented by means of the following relation:

(12) $$\frac{d\bar{M}}{d\eta }=-m_{0}\frac{d\rho \left(\mu ,\xi ,\eta \right)}{d\eta }$$

This relation will be used to validate our theoretical model based on the empirical data which will be presented in Section 3.

## 3. Results and Discussion

**Figure 1:**
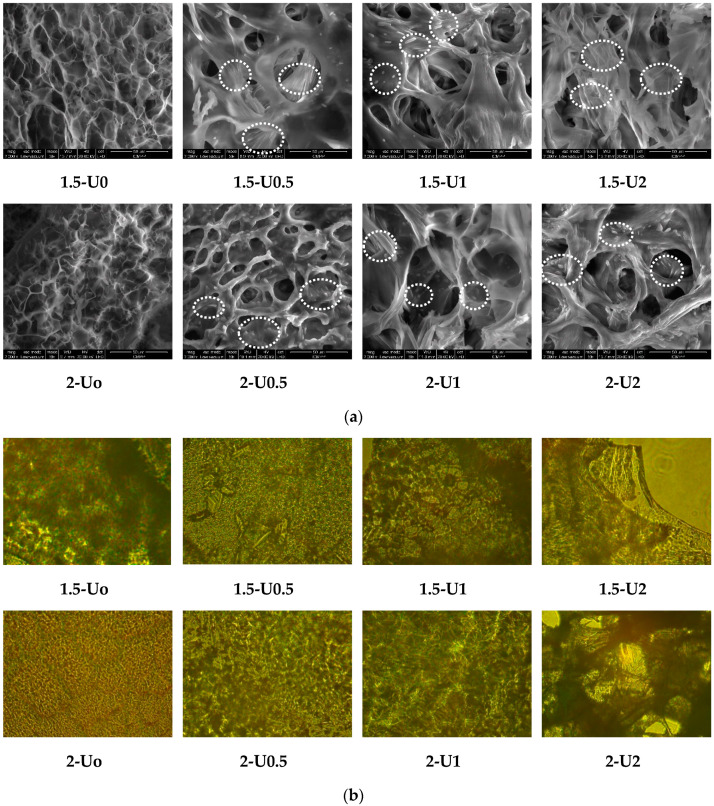
Representative (**a**) SEM (scale bar: 50 µm) and (**b**) polarized light microscopy (POM) (magnification: 400×) images of the 1.5-Ux and 2-Ux formulations. The crystals in the SEM images were marked with circles.

In view of modeling the urea release characteristics, eight formulations based on chitosan, salicylaldehyde and urea (Table 1) were prepared applying the procedure of the in situ hydrogelation described in the Experimental section. It should be remarked that the in situ procedure allowed for efficient encapsulation of a large amount of fertilizer [29,30,31,32]. The formulations were firstly investigated by scanning electron microscopy (SEM) measurements to observe the influence of both the crosslinking density and urea content on their morphology. As can be seen in Figure 1a, the formulations were porous. Compared to the blank matrix (1.5-U0, 2-U0), the formulations displayed larger pores (approx. 50 µm compared to approx. 25 µm) and visibly thicker pore walls. Moreover, in the pore walls, acicular crystals can be distinguished, characteristic of the urea crystals [33]. Compared to the scale bar, their size can be appreciated at the micrometric level. On the other hand, considering the large amount of urea compared to the matrix amount (i.e., half, equal or double), the fraction of visible micrometric crystals is quite low. This enables the visualization of a large fraction of urea crystals encapsulated at the sub-micrometric level and even at the nano-metric level. As expected, the density of the micrometric crystals seems to increase along with urea content in formulation, a feature also observed for such systems with content of bioactive components [34]. This observation was further supported by polarized light microscopy (POM) which displayed more homogeneous birefringent textures with a lower content of urea and crystalline shapes of a higher dimension as the urea content increased (Figure 1b). The birefringent crystalline shapes were attributed to the urea sub-micrometric and micrometric crystals, encapsulated in the chitosan hydrogel matrix by physical forces, developed due to the strong polycationic nature of chitosan in hydrogel state [35]. The continuous birefringence with a particular banded texture was correlated with the layered supramolecular ordering of the hydrogels [36,37].

When discussing the theoretical models used for drug-release mechanisms in the literature the homogeneity assumption in its various forms (homogenous kinetic space, law of mass etc.) is at their core. The functionality of such a hypothesis allowed the development of a class of differentiable models in the description of drug release dynamics in such systems. However, biological systems are nowadays understood as inherently non-differential (fractal). Specifically, in the microenvironments where any drug molecules with membrane interface, metabolic enzymes or pharmacological receptors are unanimously recognized as unstirred, space-restricted, heterogeneous and geometrically fractal. It is thus necessary to define a new class of models, this time non-differentiable, in describing biological system dynamics and particularly drug release dynamics in such systems. Usually, such an approach is known as Fractal Pharmacokinetics (PK) and implies the use of fractional calculus, expanding on the notion of dimension. This complex analysis allowed the modeling of processes such as drug dissolution, absorption, distribution, and kinetics with bio-molecular reactions. Our mathematical approach, in the context of “compartmental analysis”, presents itself as a new method for describing drug release dynamics in complex systems (evidently discarding fractional derivative and other standard “procedures” used in PK), considering the proposal that drug release dynamics can be described through continuous but non-differentiable curves (multifractal curves). Then, instead of “working” with a single variable described by a strict, non-differentiable function, it is possible to “operate” only with approximations of these mathematical functions, obtained by averaging them on different scale resolutions.

**Figure 2:**
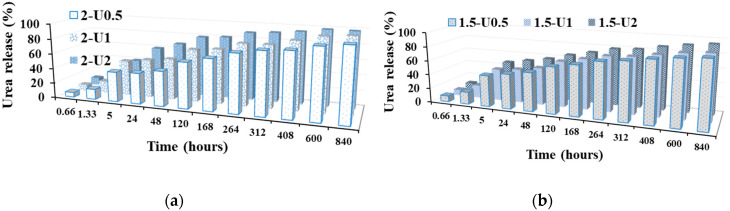
Graphical representation of the urea release from **1.5-Ux** (**b**) and **2-Ux** (**a**) formulations.

The graphical representation of the urea release from the understudy formulations is depicted in Figure 2. As can be seen, the urea release advanced in three stages and was significantly affected by the encapsulation pathway: (1) a burst release in the first 5 h (release up to 46% of urea), (2) a slower release in the next 11 days (up to 75% released urea) and (3) a continuous slow release in the next 23 days (almost all urea was released in the water medium).

From the 2-U2 and 1.5-U2 samples containing larger urea crystals, the release occurred faster, while from the other samples in which the urea was encapsulated as smaller crystals, the release produced slower. Moreover, the samples with a higher crosslinking degree (1.5-Ux) appeared to release slightly faster compared to the ones with a lower crosslinking degree (2-Ux).

**Figure 3:**
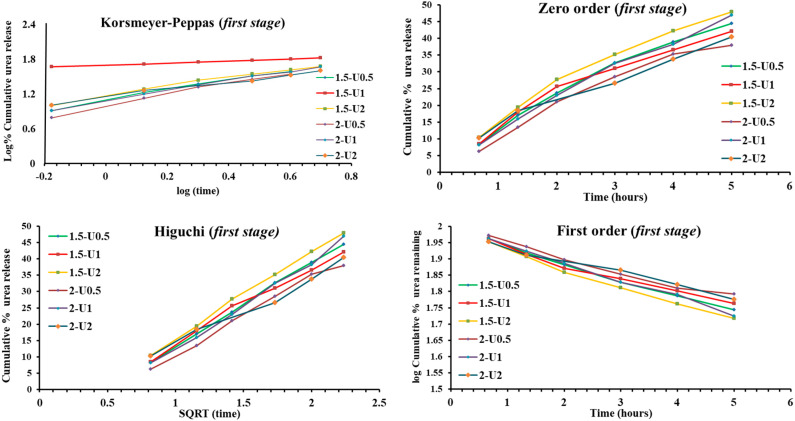

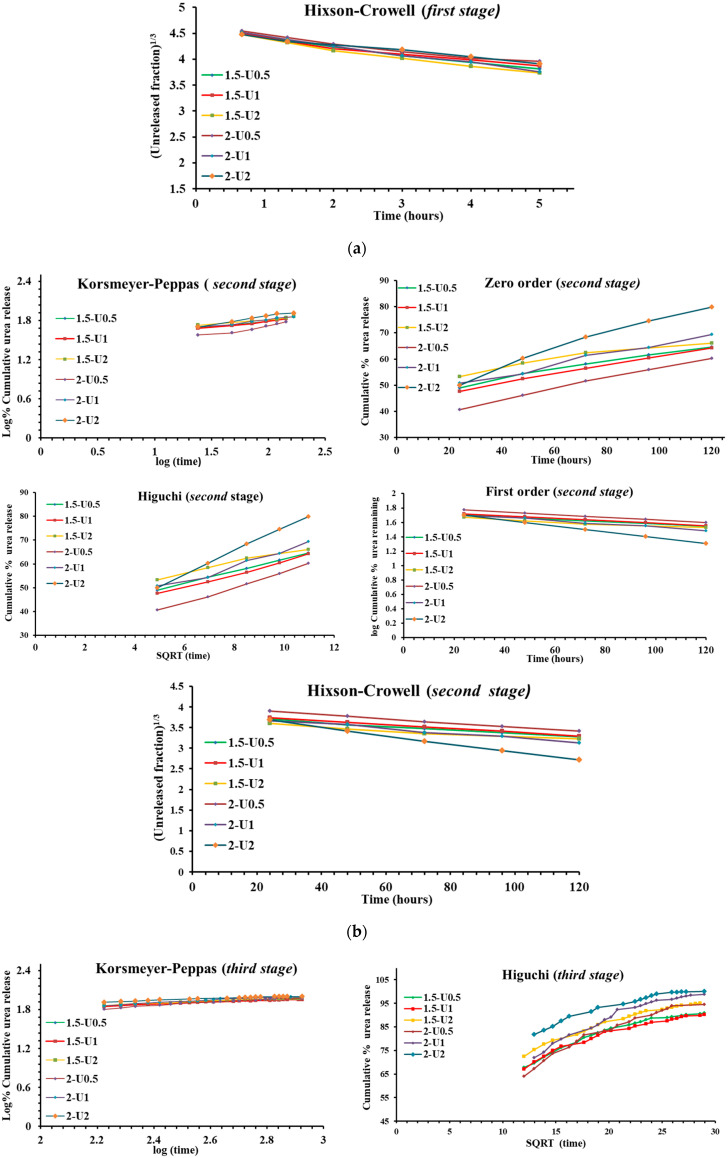

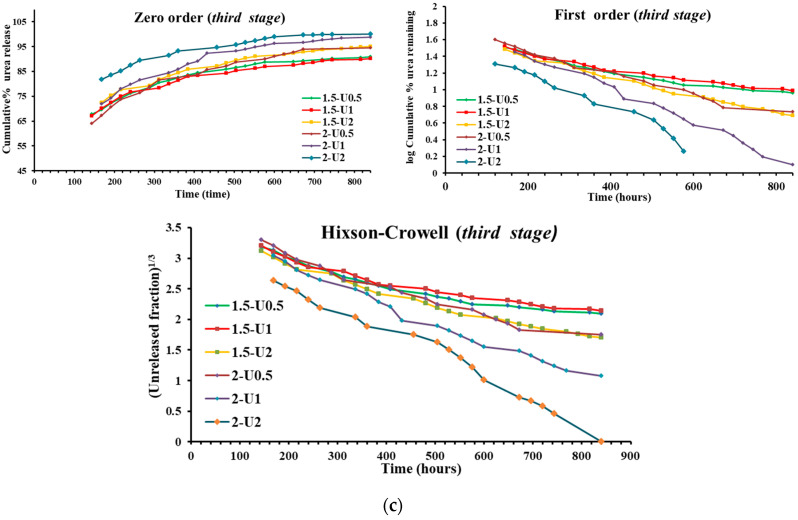
Linear forms of the all five mathematical models applied for the release of urea from 1.5-Ux and 2-Ux in (**a**) *first stage*, (**b**) *second stage* and (**c**) *third stage.*

In order to understand the kinetics release of the urea on each of the three stages, from 1.5-Ux and 2-Ux samples, the release data were fitted on the mathematical equations of the Korsmeyer–Peppas, Zero order, First order, Higuchi and Hixson–Crowell (Figure 3a–c). As can be seen in Figure 3a,b, the obtained in vitro release data proved a good fitting in the *first stage* (Figure 3a) and *second stage* (Figure 3b) on all five mathematical models. This good fitting indicates that the urea release mechanism is controlled by both dissolution and diffusion through the hydrogel matrix. Considering the morphology of the urea release systems, this mechanism correlates well with the faster dissolution of the micrometric crystals in the first stage, less anchored into the matrix, followed by the submicrometric ones in the second stage. In the *third second stage*, except with Korsmeyer –Peppas, the fitting of all mathematical models failed for almost all the samples (Figure 3c) indicating that heterogeneous erosions of the matrix occurred, which favored the release of the urea encapsulated at the nanometric level or even the molecular level, and were very well anchored into the matrix.

**Figure 4:**
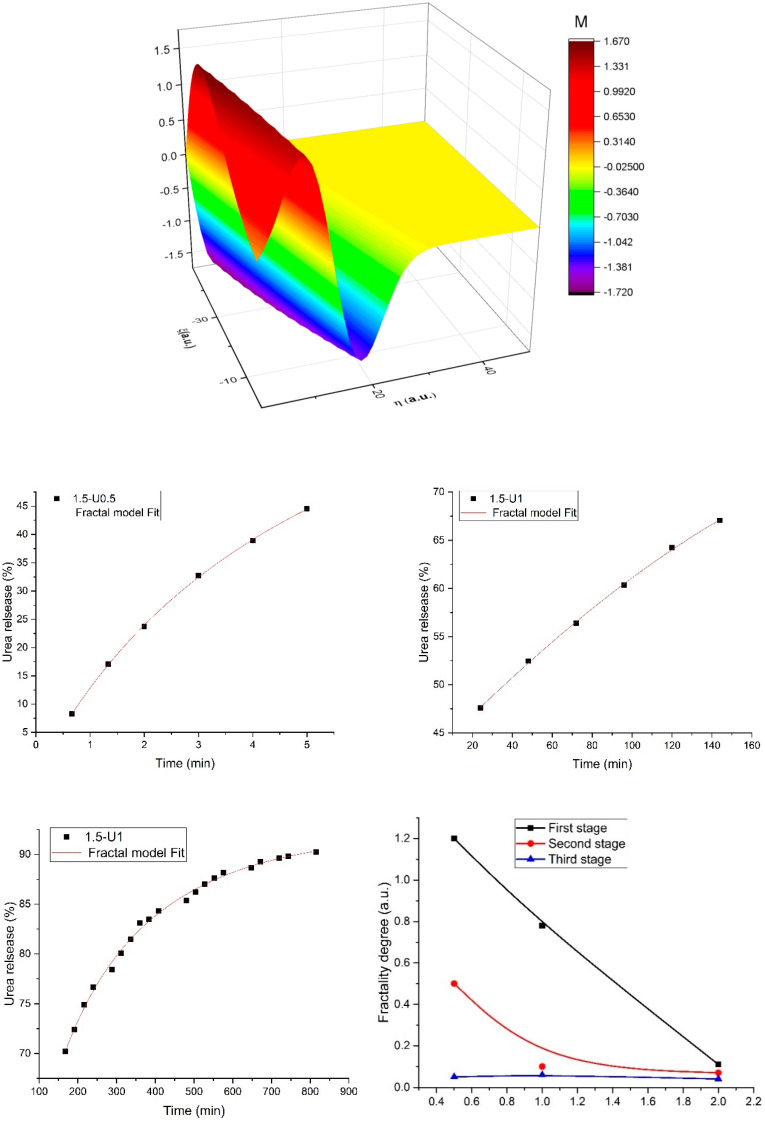
3D representation of the urea release mass evolution in time and the theoretical fit of each of the expansion stages for the 1.5-U1 case and the fractality degree evolution with the amounts of encapsulated urea.

To further understand the forces which drive the urea release, the multifractal model presented in Section 2.6 was calibrated on the empirical data presented in the previous section. In this case, the evaluation of the release kinetics has been conducted through Equation (12). In Figure 4, the 3D representation of the release mass variation in time and space and the fit of the experimental data using our model are presented. The model was calibrated [22,23,24] to fit the empirical data presented in the previous section. It can be observed that the multifractal model accurately predicts the behavior seen empirically with a steep increase for a short moment of time and a saturation plateau for considerably longer periods. The fitting when using the multifractal functions allowed the determination of the fractal degree [22,23] for each stage of the urea release scenario. The multifractal model worked at each time-scale as the inherent characteristic of the model was the possibility to transcend various scales in the framework of the same mathematical apparatus. It was observed that in the first release stage the fractality of the system was high, which meant that the release was a highly energetic one. The fractality decreased as the release advanced in time (second stage), a fact which reflects a decrease overall in the urea mass released. It should be noted that in the third stage, where the fractality degree is small, there is no significant dependence on the amount of initio urea percentage, meaning that with a slow release behavior the initial values do not affect the late time-scale behavior.

## 4. Conclusions

A number of formulations were prepared by in situ dispersion of different amounts of urea into hydrogels based on chitosan and salicylaldehyde eco-reagents in different molar ratios. SEM and POM indicated that urea was encapsulated in the form of crystals of different sizes: microcrystals, submicrometric crystals and even at a molecular level. The in vitro release data showed that urea release took place in three different stages during a 35-day period, corresponding to the different dissolution rate functions of the crystal size: (i) a faster release was favored by the rapid dissolution of the bigger crystals which were less anchored into the matrix in the first stage, followed by (ii) a slower release of the smaller crystals better anchored in the second stage, and further by (iii) the slower release of the smallest crystals during the third stage when erosion of the matrix occurred. A theoretical multifractal model has been fitted with the empirical data of the urea release from the formulations. The calibration of the theoretical multifractal model entirely confirmed this release profile, suggesting this simple model as an important tool for understanding the morphology–release relationship of the complex release systems. These good results encourage the further application of this model on other fertilizer release systems and even others such as drug release systems. The advantages of this multifractal approach need to be viewed as a more general implementation, not being directly related to one particular drug–polymer matrix. Through the scale resolution parameter, the model can navigate and describe different configurations for the drug release mechanisms.

## Figures and Tables

**Table 1:** The compositions of the urea release systems and their codes.

Code	NH_2_/CHO Molar Ratio	Chitosan (mg)	SA (mg)	Matrix (mg)	Urea		Formulation (mg)
					(mg)	(%)	
1.5-U0	1.5/1	100	41	141	0	0	141
1.5-U0.5	1.5/1	100	41	141	70.5	33	211.5
1.5-U1	1.5/1	100	41	141	141	50	282
1.5-U2	1.5/1	100	41	141	282	66	423
2-U0	2/1	100	31	131	0	0	131
2-U0.5	2/1	100	31	131	65	33	196
2-U1	2/1	100	31	131	131	50	262
2-U2	2/1	100	31	131	262	66	393

## Supplementary Materials

For details on the multifractal model used for assessing urea release from chitosan based formulations please check our supplementary material at https://www.mdpi.com/2073-4360/12/6/1264/s1.
