# Supplementary material for: A Theoretical Multifractal Model for Assessing Urea Release from Chitosan Based Formulations
Source: Polymers (Basel). 2020 Jun 1;12(6):1264. doi: 10.3390/polym12061264 (PMC7362081; doi:10.3390/polym12061264)
Supplement: Supplementary file 1 [file polymers-12-01264-s001.pdf]

polymers

Supplementary Materials

A theoretical multifractal model for assessing urea release from chitosan based formulations

Manuela Maria Iftime[1], Stefan Andrei Irimiciuc[2], Maricel Agop[3], Marin Angheloiu[4], Lacramioara Ochiuz[5*] and Decebal Vasincu[5]

[1]Romanian Academy of Sciences, Petru Poni Institute of Macromolecular Chemistry, 41A Grigore Ghica Voda Alley, 700487, Iasi, Romania
[2]National Institute for Laser, Plasma and Radiation Physics – NILPRP, 409 Atomistilor Street, Bucharest, Romania
[3] Department of Physics, "Gh. Asachi" Technical University of Iasi, Iasi 700050, Romania
[4]Center for Services and Research in Advanced Biotechnologies, Calugareni, Sanimed International Impex SRL, Road Bucuresti-Magurele, no. 70 F, sector 5 Bucharest, Romania
5 University of Medicine and Farmacy Grigore T. Popa Iasi, Iaşi, Romania

Considering that the dynamics of structural units of the polymer-drug complex systems takes place on continuous and nondifferentiable curves (multifractal-curves) we will describe these dynamics through the scale covariance derivative (for details see [19,20]);

$$\frac{\hat{d}}{dt} = \partial_t + \hat{V}^l \partial_l + \frac{1}{4}(dt)^{\left[\frac{2}{f(\alpha)}\right]-1} D^{lp} \partial_l \partial_p \tag{S1}$$

where

$$\hat{V}^l = V_D^l - V_F^l$$
$$D^{lp} = d^{lp} - i\hat{d}^{lp}$$
$$d^{lp} = \lambda_+^l \lambda_+^p - \lambda_-^l \lambda_-^p \tag{S2}$$
$$\hat{d}^{lp} = \lambda_+^l \lambda_+^p + \lambda_-^l \lambda_-^p$$
$$\partial_t = \frac{\partial}{\partial t}, \partial_l = \frac{\partial}{\partial x^l}, \partial_l \partial_p = \frac{\partial}{\partial x^l}\frac{\partial}{\partial x^p}, i = \sqrt{-1}, l, p = 1,2,3$$

In the above – written relations, x^l is the fractal spatial coordinate, t is the non – fractal time having the role of an affine parameter of the motion curves, $\hat{V}^l$ is the complex velocity, V_D^l is the differentiable velocity independent on the scale resolution dt, V_F^l is the non – differentiable velocity dependent on the scale resolution, D_F, the fractality dimension, $f(\alpha)$ is the singularity spectrum of order α of fractal dimension, α is the singularity index, D^{lp} is the constant tensor associated with the differentiable – non – differentiable transition, $\lambda_+^l(\lambda_+^p)$ is the constant vector associated with the backward differentiable – non – differentiable drug release processes and $\lambda_-^l(\lambda_-^p)$ is the constant vector associated with foreword differentiable – non – differentiable drug release processes. There are many modes, and thus a varied selection of definitions of fractal dimensions: more precisely, the fractal dimension in the sense of Kolmogorov, the fractal dimension in the sense of Hausdorff – Besikovici etc. [21,23,24] Selecting one of these definitions and operating in the drug release dynamics, the value of the fractal dimension must be constant and arbitrary for the entirety of the dynamical analysis. For example, it is regularly found $D_F < 2$ for drug release correlative processes, $D_F > 2$ for drug release non – correlative processes etc. In such a conjecture, through $f(\alpha)$ it is possible to identify not only the "areas" of the drug release dynamics that are characterized by a certain fractal dimension, but also the number of "areas" whose fractal dimensions are situated in an interval of values. More than that, through the singularity spectrum $f(\alpha)$ it is possible to identify classes of universality in the drug release dynamics laws, even when regular or strange attractors have different aspects.

For Markov – type stochastic processes [21,23,24]

$$\lambda_+^i \lambda_+^l = \lambda_-^i \lambda_-^l = 2\lambda\delta^{il} \tag{S3}$$

and for

$$f(\alpha) \equiv D_F \tag{S4}$$

where λ is a specific coefficient associated to the fractal – non – fractal transition of drug release processes and δ^{il} is Kronecker's pseudo – tensor, then the scale covariant derivative (S1) becomes:

$$\frac{\hat{d}}{dt} = \partial_t + \hat{V}^l \partial_l - i\lambda (dt)^{\left[\frac{2}{D_F}\right]-1} \partial_l \partial^l \tag{S5}$$

In the particular case of motions on Peano – type curves, which implies $D_F = 2$, the scale covariant derivative (S5) takes the standard form from the Scale Relativity Theory [24]:

$$\frac{\hat{d}}{dt} = \partial_t + \hat{V}^l \partial_l - iD\partial_l \partial^l \tag{S6}$$

where $\lambda \equiv D$ is the diffusion coefficient associated to fractal – non – fractal transition of drug release processes. Therefore, this model, generalizes all the results of Nottale's theory (i.e. Scale Relativity Theory). [24]

Now, accepting the functionality of the scale covariance principle, i.e. applying the operator (S1) to the complex velocity fields (S2), in the absence of any external constraint, the motion equations (i.e. the geodesics equation on a multifractal space) takes the following form:

$$\frac{\hat{d}\hat{V}^i}{dt} = \partial_t \hat{V}^i + \hat{V}^l \partial_l \hat{V}^i + \frac{1}{4}(dt)^{\left[\frac{2}{f(\alpha)}\right]-1} D^{lk} \partial_l \partial_k \hat{V}^i = 0 \tag{S7}$$

This means that the multifractal acceleration, $\partial_t \hat{V}^i$, the multifractal convection, $\hat{V}^l \partial_l \hat{V}^i$ and the multifractal dissipation $D^{lk} \partial_l \partial_k \hat{V}^i$ make their balance in any point of the multifractal curve. Particularly, for (S3) and (S4), the motion equation (S7) becomes:

$$\frac{\hat{d}\hat{V}^i}{dt} = \partial_t \hat{V}^i + \hat{V}^l \partial_l \hat{V}^i - i\lambda (dt)^{\left[\frac{2}{D_F}\right]-1} \partial_l \partial^l \hat{V}^i = 0 \tag{S8}$$

Now, separating the drug release dynamics on scale resolutions (the differentiable and non – differentiable scale resolutions), (S7) becomes:

$$\begin{gathered} \partial_t V_D^i + V_D^l \partial_l V_D^i - V_F^l \partial_l V_F^i + \frac{1}{4}(dt)^{\left[\frac{2}{f(\alpha)}\right]-1} D^{lk} \partial_l \partial_k V^i = 0 \\ \partial_t V_F^i + V_D^l \partial_l V_D^i + V_D^l \partial_l V_F^i - \frac{1}{4}(dt)^{\left[\frac{2}{f(\alpha)}\right]-1} D^{lk} \partial_l \partial_k V_F^i = 0 \end{gathered} \tag{S9}$$

while (S8) takes the form:

$$\begin{gathered} \partial_t V_D^i + V_D^l \partial_l V_D^i - \left[V_F^l + \lambda (dt)^{\left[\frac{2}{f(\alpha)}\right]-1} \partial^l \right] \partial_l V_F^i = 0 \\ \partial_t V_F^i + V_D^l \partial_l V_F^i + \left[V_F^l + \lambda (dt)^{\left[\frac{2}{f(\alpha)}\right]-1} \partial^l \right] \partial_l V_D^i = 0 \end{gathered} \tag{S10}$$

For irrotational motions of the polymer drug structural units, the complex velocity fields (S2) become:

$$\hat{V}^i = -2i\lambda (dt)^{\left[\frac{2}{f(\alpha)}\right]-1} \partial^i \ln \Psi \tag{S11}$$

where

$$\chi = -2i\lambda(dt)^{\left[\frac{2}{f(\alpha)}\right]-1} ln\,\Psi \tag{S12}$$

is the complex scalar potential of the complex velocity fields (S2) and Ψ is the function of states. In these conditions, substituting (S11) in (S8) and using the mathematical procedures from [19,20], the geodesics equation (motion equations) (S8) takes the form of the multifractal Schrödinger – type equation:

$$\lambda^2(dt)^{\left[\frac{4}{f(\alpha)}\right]-2}\partial^l\partial_l\Psi + i\lambda(dt)^{\left[\frac{2}{f(\alpha)}\right]-1}\partial_t\Psi = 0 \tag{S13}$$

Therefore, for the complex velocity fields of the form (S11), the dynamics of the polymer-drug structural units are described through Schrödinger "regimes" of multifractal type (i.e. Schrödinger – type equations at various scale resolutions).

Moreover, if we choose Ψ of the form:

$$\Psi = \sqrt{\rho}e^{is} \tag{S14}$$

where $\sqrt{\rho}$ is the amplitude and s is the phase, then the complex velocity fields (S11) take the explicit form:

$$\hat{V}^i = 2\lambda(dt)^{\left[\frac{2}{f(\alpha)}\right]-1}\partial^i s - i\lambda(dt)^{\left[\frac{2}{f(\alpha)}\right]-1}\partial^i \ln\rho \tag{S15}$$

which enables us to define the velocity fields:

$$V_D^i = 2\lambda(dt)^{\left[\frac{2}{f(\alpha)}\right]-1}\partial^i s \tag{S16}$$

$$V_F^i = i\lambda(dt)^{\left[\frac{2}{f(\alpha)}\right]-1}\partial^i \ln\rho \tag{S17}$$

By (S14), (S16) and (S17) and using the mathematical procedures from [19,20], the geodesics equation (S13) reduces to the multifractal hydrodynamic – type equations:

$$\partial_t V_D^i + V_D^l\partial_l V_D^i = -\partial^i Q \tag{S18}$$

$$\partial_t\rho + \partial_l(\rho V_D^l) = 0 \tag{S19}$$

with Q the specific multifractal potential:

$$Q = -2\lambda^2(dt)^{\left[\frac{4}{f(\alpha)}\right]-2}\frac{\partial^l\partial_l\sqrt{\rho}}{\sqrt{\rho}} = -V_F^i V_F^i - \frac{1}{2}\lambda(dt)^{\left[\frac{2}{f(\alpha)}\right]-1}\partial_l V_F^l \tag{S20}$$

The equation (S18) corresponds to the specific momentum conservation law of multifractal type, while equation (S19) corresponds to the states density conservation law of multifractal type. The specific multifractal potential (S20) implies the specific multifractal force:

$$F^i = -\partial^i Q = -2\lambda^2(dt)^{\left[\frac{4}{f(\alpha)}\right]-2}\partial^i\frac{\partial^l\partial_l\sqrt{\rho}}{\sqrt{\rho}} \tag{S21}$$

which is a measure of the multifractality of the motion curves.

Therefore, for the complex velocity fields of the form (S13), the dynamics of the polymer-drug structural units are described through hydrodynamic "regimes" of multifractal type (i.e. hydrodynamic equations at various scale resolutions).
